# Asrij/OCIAD1 contributes to age-associated microglial activation and neuroinflammation in mice

**DOI:** 10.3389/fnagi.2025.1674136

**Published:** 2025-10-10

**Authors:** Prathamesh Dongre, Madhu Ramesh, Thimmaiah Govindaraju, Maneesha S. Inamdar

**Affiliations:** ^1^Molecular Biology and Genetics Unit, Jawaharlal Nehru Centre for Advanced Scientific Research (JNCASR), Bangalore, Karnataka, India; ^2^Bioorganic Chemistry Laboratory, New Chemistry Unit, Jawaharlal Nehru Centre for Advanced Scientific Research (JNCASR), Bangalore, Karnataka, India; ^3^Institute for Stem Cell Science and Regenerative Medicine (BRIC inStem), Bangalore, Karnataka, India

**Keywords:** Asrij/OCIAD1, aging, microglia, astrocytes, lipopolysaccharide, inflammatory signaling, neuroinflammation

## Abstract

Aging is characterized by chronic low-grade neuroinflammation, which increases the risk of neurodegenerative disorders. Neuroinflammation, driven by the activation of astrocytes and microglia, underlies age-associated cognitive deficits. Amplified neuroinflammatory responses to immune challenges are attributed to microglial activation in the aged brain. Despite extensive clinical and experimental evidence linking neuroinflammation to aging, the molecular players that control age-associated neuroinflammatory responses in the brain are not fully understood. Genome-wide association studies (GWAS), proteomics, and transcriptomic datasets have revealed that Asrij/OCIAD1 is a novel aging and Alzheimer’s disease (AD)-associated factor. Asrij levels are increased in patients with AD and are known to promote amyloid-beta (Aβ) pathology and microglia-mediated neuroinflammation, which are associated with cognitive dysfunction in AD. Increased levels of Asrij are also reported in the brains of aged wild-type (WT) mice; however, whether this may promote neuroinflammation or be a protective response during aging is not known. To test this, we used young and aged WT and *asrij* KO mice and showed that normal aging is associated with increased microgliosis and astrocyte activation in WT mice. While young *asrij* KO mice do not display any differences in glial activation, aged KO mice have reduced microglial and astrocytic activation compared to aged WT mice. This is accompanied by reduced levels of pro-inflammatory mediators and downregulation of STAT3 and NF-κB signaling in the cortex and hippocampus of aged *asrij* KO mice. Additionally, *asrij* depletion inhibits LPS-induced microglial activation and neuroinflammation in aged mice. This indicates that Asrij is essential for the neuroinflammatory responses in the brains of aged mice. We propose that identifying pharmaceutical modulators of Asrij could provide novel means to control microglial activation and neuroinflammation during normal aging.

## Introduction

Aging is a major risk factor for the development of neurodegenerative disorders and represents a significant socioeconomic burden worldwide. Aging induces several molecular, cellular, and functional alterations in the brain that drive cognitive decline (Banks et al., 2021; López-Otín et al., 2013). These include altered synaptic plasticity and neuronal circuits, loss of myelination, increased vascular permeability, and neuroinflammation (Bieri et al., 2023). Among these factors, chronic low-grade neuroinflammation is increasingly recognized as a primary driver of age-associated behavioral deficits (Andronie-Cioara et al., 2023; Costa et al., 2021; Muzio et al., 2021; Zhang et al., 2024). Neuroinflammation is defined as an immune response in the central nervous system (CNS) that involves the production of inflammatory mediators by glia and immune cells (Heneka et al., 2024; Leng and Edison, 2021). The increased inflammatory potential of the aged brain is mainly attributed to microglia, the predominant brain-resident innate immune cells and a major source of inflammatory cytokines (Angelova and Brown, 2019; Antignano et al., 2023). Aged microglia exist in a ‘primed’ state wherein they produce exaggerated inflammatory responses to immune stimuli (Angelova and Brown, 2019; Candlish and Hefendehl, 2021). This underlies the increased susceptibility of older individuals to cognitive dysfunction following events that elicit neuroinflammation, such as infection, surgery, and brain injury (Niraula et al., 2017; Norden et al., 2015). Molecular regulators that influence neuroinflammatory responses in the brain during aging are less explored.

A meta-analysis of genome-wide association studies (GWAS) identified Asrij/Ovarian Carcinoma Immunoreactive Antigen Domain-containing protein 1 (OCIAD1) as one of the genes associated with age-related disorders (Chignon et al., 2020). Asrij is a multifunctional protein that plays an essential role in stem cell maintenance, hematopoiesis, immunity, and cancer (Khadilkar et al., 2014; Kulkarni et al., 2011; Praveen et al., 2020; Sinha et al., 2013). It localizes predominantly to the mitochondria and regulates mitochondrial homeostasis and cellular signaling to maintain blood cell homeostasis and immune responses in *Drosophila* and mice (Khadilkar et al., 2017; Ray et al., 2021; Sinha et al., 2019, 2022). Integrative analysis of proteomics and transcriptomics datasets revealed increased Asrij levels in patients with Alzheimer’s disease (AD) and mouse models. *In vitro* studies have shown that Asrij promotes mitochondria-mediated neuronal apoptosis in AD (Li et al., 2020). Importantly, *asrij* deletion in the APP/PS1 mouse model of AD improves cognition, ameliorates Aβ pathology, and dampens microglia-mediated neuroinflammation. Furthermore, AD microglia depleted of Asrij show reduced activation of inflammatory signaling pathways and fail to attain a state of disease-associated microglia (DAM), highlighting the critical role of Asrij in facilitating microglial pro-inflammatory responses (Dongre et al., 2025). Although it is clear that Asrij promotes neurodegeneration and AD progression, the significance of increased Asrij levels during normal aging is not understood. This study investigates whether Asrij contributes to microglial activation and neuroinflammation in wild-type aged mice, a role similar to that observed in AD. We demonstrate that the aged *asrij* KO mice exhibit reduced signatures of neuroinflammation. Moreover, *asrij* depletion impairs LPS-induced microglial activation and pro-inflammatory responses, indicating that Asrij is a positive regulator of age-associated neuroinflammation.

## Methods

### Maintenance and genotyping of mice

Generation and validation of the *asrij* floxed and KO mice have been described previously (Sinha et al., 2019). To generate constitutive whole-body *asrij* KO mice, homozygous *asrij* floxed (*asrij*^flox/flox^) mice were bred with hemizygous CMV-Cre^+^ (Strain #006054) mice to obtain heterozygous *asrij* KO mice (*asrij*^+/flox^; CMV-Cre^+^). These were then crossed with homozygous *asrij* floxed mice to generate homozygous *asrij* KO mice (*asrij*^−/−^; CMV-Cre^+^). For genetic identification of floxed and KO mice, DNA was isolated from tail clippings using a crude NaOH extraction protocol. Genotyping PCR was performed using the primers Arj_F1 (5′- GGAGAATTGCGGCGCTCTTCTCC -3′) and Arj_R1 (5′- CCATCCATCCCTCTCCACTGG −3′) to amplify the wild-type locus (608 bp) and the floxed locus (681 bp), and primers Arj_F2 (5′- ATGAAGCAGTGTCTTGGGATTGC -3′) and Arj_R1 (5′- CCATCCATCCCTCTCCACTGG −3′) to detect the excised copy (535 bp). Young (4-month-old) and aged (24-month-old) *asrij* floxed and KO mice were bred separately as homozygous stocks for all experiments. Age-matched male mice were used in this study. Mice were maintained at the Jawaharlal Nehru Centre for Advanced Scientific Research (JNCASR) animal facility in an individually ventilated cage (IVC) system under ambient temperature (23 ± 1.5 °C), a 12-h light–dark cycle, and with *ad libitum* standard rodent chow diet and water. Mice were euthanized using cervical dislocation. All mouse experiments and protocols were approved by the JNCASR Institutional Animal Ethics Committee (Project #MSI005) and conducted in compliance with the guidelines and regulations.

### Cell culture

Mouse N9 microglial cells were cultured in DMEM (Gibco, Thermo Fischer Scientific, USA; #12800–017) containing 1X GlutaMAX (Gibco, Thermo Fischer Scientific, USA; #35050–061) and 10% Fetal Bovine Serum (Gibco, Thermo Fischer Scientific, USA; #1010270106) at 37 °C in a humidified 5% CO_2_ incubator. For the *in vitro* LPS treatment, 2 × 10^5^ cells were plated and cultured overnight. The next day, microglia were stimulated with 100, 250, and 500 ng/mL LPS (Sigma-Aldrich, USA; #L2630) for 12 h at 37 °C and then harvested for lysate preparation and immunoblotting.

### Acute *in vivo* LPS treatment of mice

Aged (24-month-old) *asrij* floxed and KO mice were intraperitoneally injected with 0.5 mg/kg LPS (Sigma-Aldrich, USA; #L2630) or saline as per the previously published procedure (Cheng et al., 2021). Whole brains were collected 24 h after the injection. The left hemisphere was processed for cryosectioning and immunofluorescence, while the right hemisphere was used for qPCR and immunoblotting.

### Immunohistochemistry

Processing of the mouse brain samples and immunohistochemistry were performed as described previously (Dongre et al., 2025). Mice were euthanized by cervical dislocation, and their brains were rapidly isolated and bisected. The left hemisphere was fixed with 4% paraformaldehyde (PFA) at 4 °C for 48 h. Hippocampi and cortices from the right hemisphere were micro-dissected and flash-frozen at −80 °C for protein and RNA extraction. Fixed hemibrains were cryoprotected in 30% sucrose at 4 °C for 48 h, embedded in PolyFreeze medium (Sigma-Aldrich, USA; #SHH0026), and sectioned coronally (30 μm) using a cryostat (Leica, #CM3050S). Sections were collected on 0.3% gelatin-coated slides and stored at −80 °C. For immunofluorescence, the slides were thawed to room temperature (RT), washed with PBS + 0.05% sodium azide, and permeabilized in 0.3% Triton X-100 (Sigma-Aldrich, USA; T8787) for 1 h at RT. Sections were blocked with 4% FBS (Gibco, Thermo Scientific, USA; #1010270106) for 1 h at RT and then incubated overnight at 4 °C with the following primary antibodies: GFAP (Cloud Clone Corporation, USA; #PAA068Mu02) and IBA1 (Cell Signaling Technology, USA; #17198S). After washing, the sections were incubated for 1 h at RT with Alexa Fluor 488 conjugated anti-rabbit secondary antibody (1,400, Invitrogen, USA, #A-11008), washed again, and mounted with ProLong Gold Antifade containing DAPI (Roche, Switzerland; #P36930).

### Confocal imaging and analysis

Hippocampal and cortical regions were imaged using a Zeiss LSM 880 confocal microscope (Zeiss, Germany) with 20 × and 63 × objectives at 0.5 μm z-intervals. For quantification, 20 × images were acquired from three serial sections per mouse, with four to five images per section for each region. Images were processed in Fiji/ImageJ (NIH, MD, USA), converted to 8-bit, background subtracted (rolling-ball radius, 50 pixels), and thresholded to calculate the percentage staining area. Cell numbers were quantified using the “Analyze Particle” Function. For morphological analysis of microglia and astrocytes, 63 × images were exported to Imaris (Oxford Instruments, UK) and skeletonized using the ‘Filament’ module. At least 20–25 cells per section (three sections per mouse) were analyzed. Representative images are shown as maximum intensity projections.

### Immunoblotting

Preparation of lysates and western blotting was performed as described previously (Dongre et al., 2025). Frozen hemibrains were homogenized in 500 μL lysis buffer [20 mM HEPES (pH 7.5), 150 mM NaCl, 5 mM MgCl₂, 5 mM EDTA (pH 8), 0.5% Triton X-100, 10% glycerol, 5 mM DTT, 1 mM PMSF, 10 mM NaF, 1 mM Na₃VO₄, and protease inhibitors (Sigma-Aldrich, USA)] using a tissue tearor. Lysis was performed for 4 h at 4 °C and then centrifuged at 16,000 rcf for 30 min at 4 °C. Supernatants were stored at −80 °C, and protein concentrations were measured using the Bradford assay (Bio-Rad, USA). Equal protein amounts (40 μg) were denatured in Laemmli buffer at 99 °C for 5–10 min, separated using 10–12% SDS-PAGE, and transferred to nitrocellulose membranes. Membranes were blocked with 5% skim milk in PBST (PBS + 0.1% Tween 20) for 1 h at room temperature (RT), then incubated overnight at 4 °C with primary antibodies against Asrij/OCIAD1 (Abcam; #ab91574), IBA1 (#17198S), STAT3 (#9139S), p-STAT3 (Tyr705) (#9145S), NF-κB p65 (#8242S), p-NF-κB p65 (Ser536) (#3033S), TNF-*α* (#11948S), IL-6 (#12912S) (all from Cell Signaling Technology, USA), Glyceraldehyde-3-Phosphate Dehydrogenase (GAPDH, #G9545), and α-tubulin (#T8203) (both from Sigma-Aldrich). After washing, the membranes were incubated with HRP-conjugated anti-rabbit or anti-mouse secondary antibodies (GeNei Laboratories, India) for 1 h at RT, developed with Clarity ECL reagent (Bio-Rad, USA; #1705061), and visualized on X-ray films. Band intensities were quantified in Fiji and normalized to the loading controls.

### Enzyme-linked immunosorbent assay (ELISA)

100 μg protein from hippocampal and cortical lysates was used to measure the levels of cytokines, IL-6, and TNF-α. BD OptEIA mouse IL-6 (#550950) and BD OptEIA TNF-α (#560478) (both from BD Biosciences, CA, USA) ELISA kits were used, according to the manufacturer’s instructions. Absorbance was measured using a Varioskan LUX multimode microplate reader (Thermo Fisher Scientific, MA, USA). A standard curve was generated using known concentrations and fitting a sigmoidal curve in Prism 9.0 (GraphPad, USA). The concentration of the samples was obtained by interpolation. The assay was performed in duplicate per sample for three independent biological replicates.

### RNA isolation and reverse transcription-quantitative PCR (RT-qPCR)

RNA isolation from the brain and RT-qPCR were performed as described previously (Dongre et al., 2025). Briefly, the hippocampus and cortex tissues were minced and homogenized in 1 mL ice-cold TRIzol reagent (Invitrogen, CA, USA; #15596–026). RNA isolation was performed according to the manufacturer’s instructions. cDNA was synthesized using 2 μg total RNA in the SuperScript III First-Strand Synthesis Kit (Invitrogen, USA; #18080051). RT-qPCR was performed using the SensiFAST SYBR No-ROX kit (Bioline/Meridian Life Sciences, USA; # BIO-98020) in a CFX384 real-time PCR system (Bio-Rad, USA), according to the manufacturer’s protocol. All reactions were performed in duplicate, and GAPDH was used as a loading control. Relative transcript levels were calculated and normalized using the 2^−ΔΔCT^ method. The following primers were used: iNos2 (Forward: CCCTCCTGATCTTGTGTTGGA, Reverse: CAACCCGAGCTCCTGGAA), IL-1β (Forward: CACAGCAGCACATCAACAAG, Reverse: GTGCTCATGTCCTCATCCTG), IL-18 (Forward: GACAGCCTGTGTTCGAGGATATG, Reverse: TGTTCTTACAGGAGAGGGTAGAC), TNF-*α* (Forward: GCCTCTTCTCATTCCTGCTTG, Reverse: CTGATGAGAGGGAGGCCATT), NF-κB (Forward: GCTGCCAAAGAAGGACACGACA, Reverse: GGCAGGCTATTGCTCATCACAG), Nlrp3 (Forward: AGAAGCTGGGGTTGGTGAATT, Reverse: GTTGTCTAACTCCAGCATCTG), C3 (Forward: AGCTTCAGGGTCCCAGCTAC, Reverse: GCTGGAATCTTGATGGAGACGC), C1qbp (Forward: CCACGCAACGGCAAGTTCAC, Reverse: CGGCCACGAACGAGATTCAC), P2ry12 (Forward: AAACTCGGGCCGTCTTTGA, Reverse: GACGTCAGCCATAGGGTGCT), Tmem119 (Forward: ACTACCCATCCTCGTTCCCTGA, Reverse: TAGCAGCCAGAATGTCAGCCTG), Cx3cr1 (Forward: CTGTTATTTGGGCGACATTG, Reverse: AACAGATTTCCCACCAGACC), Trem2 (Forward: CTGCTGATCACAGCCCTGTCCCAA, Reverse: CCCCCAGTGCTTCAAGGCGTCATA), Apoe (Forward: GAACCGCTTCTGGGATTACCTG, Reverse: GCCTTTACTTCCGTCATAGTGTC), Itgax (Forward: ATCCTTGTCTACCCCAGTGC, Reverse: CATCCAGGGCTAGCTGAAG), Clec7a (Forward: CCAGCTAGGTGCTCATCTACTG, Reverse: CCTTCACTCTGATTGCGGGAAAG), Axl (Forward: GGAGGAGCCTGAGGACAAAGC, Reverse: TACAGCATCTTGAAGCCAGAGTAGG), Arg1 (Forward: GGAGACCACAGTCTGGCAGTTGGA, Reverse: GGACACAGGTTGCCCATGCAGA), Il10 (Forward: CCCTTTGCTATGGTGTCCTT, Reverse: TGGTTTCTCTTCCCAAGACC), Vimentin (Forward: CGGAAAGTGGAATCCTTGCAGG, Reverse: AGCAGTGAGGTCAGGCTTGGAA), Lipocalin 2 (Forward: ATGTCACCTCCATCCTGGTCAG, Reverse: GCCACTTGCACATTGTAGCTCTG), S100a10 (Forward: GAAAGGGAGTTCCCTGGGTT, Reverse: CCCACTTTTCCATCTCGGCA), Eaat1 (Forward: CGGGATTCCTCAGGCCGGTC, Reverse: GTTCGGAGGCGGTCCAGAAACC) and Serpina3n (Forward: CAACCAGAGACCCTGAGGAAGT, Reverse: AGGACATCCTCCAGGCTGTAGT).

### Statistical analysis

Each data point in the graphs corresponds to an individual mouse. All data are expressed as mean ± standard error of the mean (SEM). Statistical significance between two groups was assessed using an unpaired two-tailed Student’s t-test. For comparisons involving more than two groups, two-way analysis of variance (ANOVA) was performed, followed by Tukey’s multiple comparison post-hoc test. All statistical analyses and graph generation were conducted using the Prism 10 software (GraphPad). A *p*-value of less than 0.05 was considered statistically significant. The following notation was used to indicate significance levels: ns— not significant, *p < 0.05, **p < 0.01, and ***p < 0.001.

## Results

### Aged *asrij* KO mice exhibit reduced glial activation and decreased neuroinflammation

Heightened neuroinflammation is a central feature of brain aging, attributed to activated microglia and astrocytes (Edler et al., 2021). Increased Asrij protein levels in the brain are apparent in aged (18-month-old) WT mice (Li et al., 2020). This correlates with the increased numbers of glia and neuroinflammation in the aged WT mice. Given that Asrij promotes microglial activation and neuroinflammation in AD, we investigated whether it plays a similar role in normal aging.

**Figure 1:**
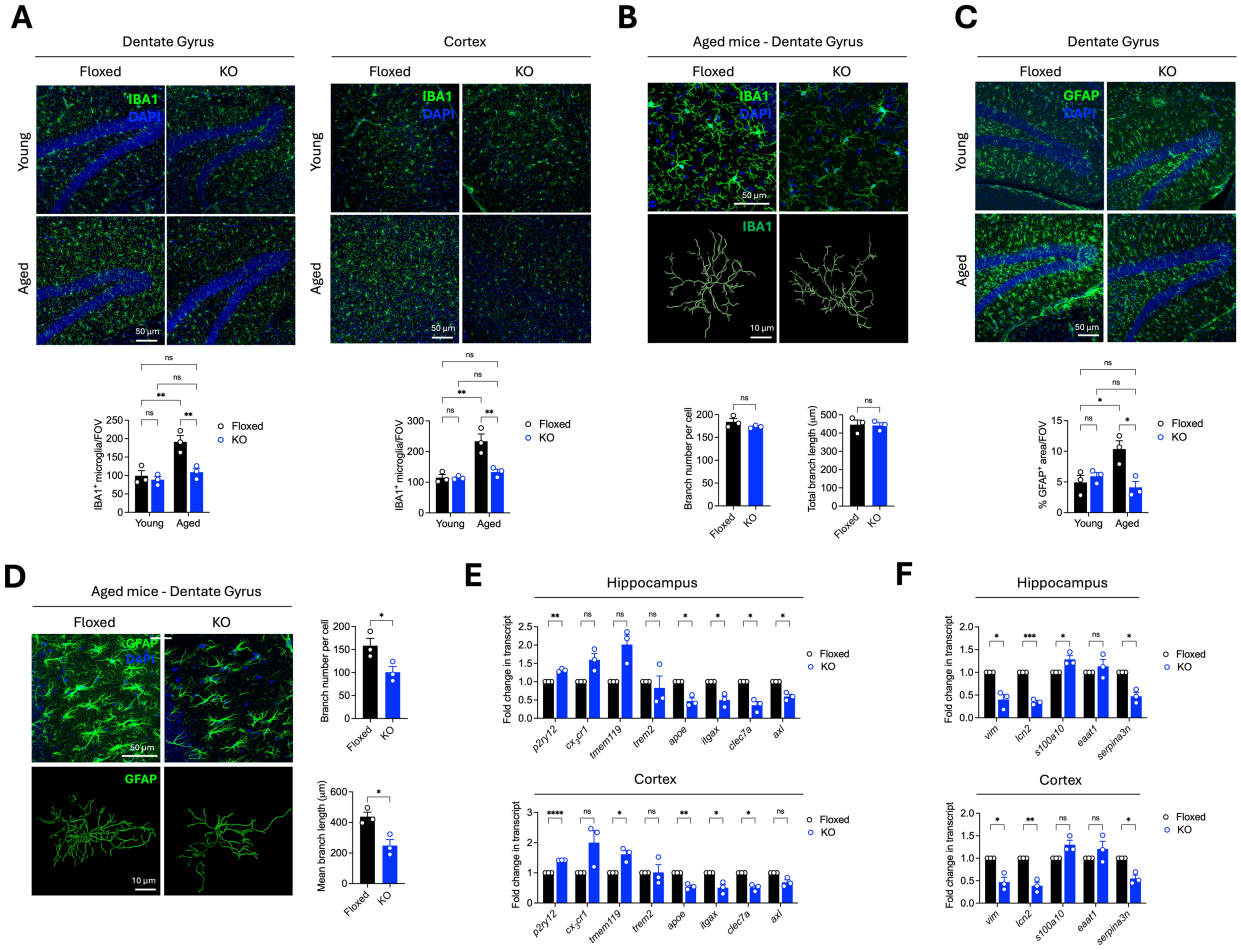
Asrij depletion results in reduced activation of microglia and astrocytes in the aged mouse brain. Young (4-month-old) and aged (24-month-old) male mice were used in the experiments. **(A)** Representative confocal images of brain sections show IBA1 staining in the dentate gyrus and cortex. Graphs show quantification of the number of microglia per field of view (FOV) and the percentage of IBA1^+^ area (*n* = 3 mice, 3 sections per mouse). **(B)** Representative images show IBA1^+^ cortical microglia and renderings of the microglial skeleton using the ‘filament’ module of Imaris. Graphs show quantification of branch number per microglia and total branch length (*n* = 3 mice, 25 cells per mouse). **(C)** Representative confocal images show GFAP staining in the dentate gyrus. The graph shows the quantification of the percentage of GFAP^+^ area (*n* = 3 mice, 3 sections per mouse). **(D)** Representative images show GFAP^+^ astrocytes in the hippocampus and Imaris-based renderings of the astrocyte skeleton. Graphs show quantification of branch number and branch length per cell (*n* = 3 mice, 25 cells per mouse). **(E)** Graphs show quantification of fold change in normalized transcript expression of microglial activation markers in the cortex and hippocampus, analyzed by RT-qPCR (*n* = 3 mice). GAPDH is used as a loading control. **(F)** Graphs show quantification of fold change in normalized transcript expression of astroglial activation markers in the cortex and hippocampus, analyzed by RT-qPCR (*n* = 3 mice). GAPDH is used as a loading control. Statistical significance between experimental groups was calculated using two-way ANOVA with Tukey’s *post hoc* test **(A,C)** and unpaired two-tailed Student’s *t*-test **(B,E,F)**. Error bars denote mean ± SEM. ns—non-significant, *p < 0.05, **p < 0.01, and ***p < 0.001.

To investigate whether Asrij impacts the maintenance of glia in the brain during normal aging, we used young (4-month-old) and aged (24-month-old) *asrij* floxed (control) and *asrij* KO mice. Immunostaining of brain cryosections showed increased numbers of IBA1^+^ microglia in aged *asrij* floxed mice than in the young *asrij* floxed mice. This finding is in agreement with the previous reports that support age-associated increases in microglial coverage in mice (Niraula et al., 2017). Young *asrij* KO mice did not exhibit changes in microglial numbers compared to young floxed mice. Interestingly, aged KO mice showed reduced microglia in the hippocampus and cortex compared to the aged control mice (Figure 1A). However, Imaris-based analysis of microglial morphology revealed no significant differences in branch length and branch number due to Asrij depletion in aged mice (Figure 1B). Thus, Asrij may be essential to maintain microglial numbers but not morphology in the aged mice.

One of the key aging-associated changes in the brain is the phenotypic shift of astrocytes to a neuroinflammatory A1-like reactive state, which is marked by increased GFAP levels (Clarke et al., 2018; Habib et al., 2020; Patani et al., 2023). As expected, we observed increased GFAP coverage in the aged control mice compared to the young control mice, indicating age-associated astrocyte activation. Young *asrij* KO mice showed equivalent levels of GFAP staining compared to young control mice. Interestingly, aged *asrij* KO mice had decreased GFAP coverage compared to aged control mice, indicating that Asrij contributes to age-related astrocyte activation (Figure 1C). Astrocyte activation is accompanied by a transition from a stellate morphology that marks the resting or homeostatic state to a hypertrophic morphology marking the reactive state (Liddelow and Barres, 2017). Hence, we also evaluated astrocyte morphology using the ‘filament’ tool of Imaris. While aged control mice displayed astrocytes with numerous branches, *asrij* KO mice exhibited reduced astrocytic branch number and branch length, suggesting decreased activation (Figure 1D).

Transcriptomic analysis of microglia and astrocytes from young and aged mice has revealed several gene expression signatures of activation (Paolicelli et al., 2022; Patani et al., 2023). Notably, aged glia show alterations in gene modules associated with tissue repair, inflammation, synaptic functions, and metabolism, among others (Candlish and Hefendehl, 2021). We used RT-qPCR to assess the expression of markers linked to microglial and astroglial activation in aged *asrij* KO mice. Aged *asrij* KO mice showed increased levels of homeostatic microglial markers (P2ry12, Tmem119, and Cx3cr1) and a concomitant increase in Disease-Associated Microglia (DAM) markers such as Clec7a, Axl, Apoe, and Itgax (Figure 1E). Similarly, aged *asrij* KO mice had reduced levels of astrocyte activation markers such as Lipocalin 2, S100a10, and Serpina3n (Figure 1F). Thus, our RT-qPCR analysis is in agreement with the imaging studies and further strengthens the findings that loss of Asrij attenuates glial activation in the aged mouse brain.

**Figure 2:**
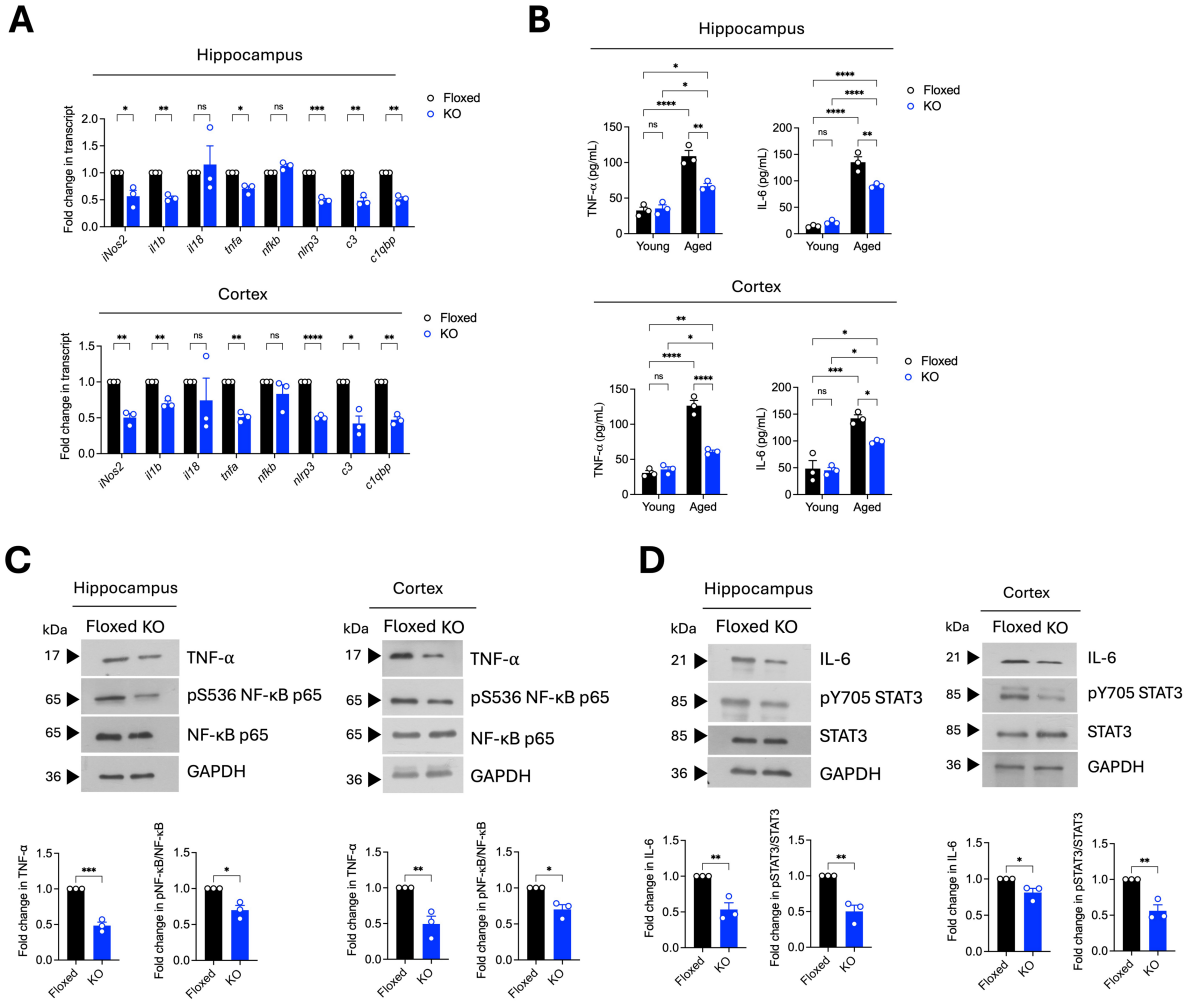
Aged *asrij* KO mice exhibit reduced neuroinflammation. Aged (24-month-old) male mice were used in the experiments. **(A)** Graphs show the quantification of the fold change in normalized transcript expression in the cortex and hippocampus, analyzed by RT-qPCR (*n* = 3 mice). GAPDH is used as a loading control. **(B)** Graphs show quantification of concentrations of TNF-*α* and IL-6 (pg/mL) in the cortex and hippocampus determined by ELISA (*n* = 3 mice). **(C)** Immunoblot analysis of TNF-α, phospho-NF-κB (Ser536), and NF-κB levels in the hippocampus and cortex (*n* = 3 mice). Graphs show the quantification of fold change in normalized protein levels. GAPDH is used as a loading control. **(D)** Immunoblot analysis of IL-6, phospho-STAT3 (Tyr705), and STAT3 levels in the hippocampus and cortex (*n* = 3 mice). The graph shows quantification of fold change in normalized protein levels. GAPDH is used as a loading control. Statistical significance between experimental groups was calculated using two-way ANOVA with Tukey’s post hoc test **(B)** and unpaired two-tailed Student’s *t*-test **(A,C,D)**. Error bars denote mean ± SEM. ns—non-significant, *p < 0.05, **p < 0.01, and ***p < 0.001.

A consequence of glial activation in the aged brain is the exaggerated production of pro-inflammatory mediators, such as cytokines and chemokines, in the brain milieu. Such hyperactive immune responses contribute to age-related neuropathology and cognitive defects in rodents (Candlish and Hefendehl, 2021; Deng et al., 2023). Given that *asrij* depletion led to reduced glial activation, we assessed the pro-inflammatory gene expression in the aged brain using RT-qPCR. Interestingly, there was a significant downregulation of pro-inflammatory genes, namely inducible nitric oxide synthase (iNos2), interleukin-1 beta (IL-1β), tumor necrosis factor (TNF-*α*), NOD-, LRR-, and pyrin domain-containing protein 3 (Nlrp3), and complement factors C3 and C1qbp in the aged *asrij* KO brain (Figure 2A). We also measured the levels of two key pro-inflammatory cytokines, TNF-α and IL-6, using enzyme-linked immunosorbent assay (ELISA). Young KO mouse brains had normal levels of TNF-α and IL-6 compared to those of young floxed mice (Figure 2B). As expected, aged control mice showed elevated levels of IL-6 and TNF-α in hippocampal and cortical homogenates compared to young control mice. While there was an increase in levels of IL-6 and TNF-α upon aging in the KO brains, this was not to the same extent as that in control aged brains (Figure 2B). This suggests that the increase in Asrij levels during normal aging may promote or sustain the proinflammatory state of the aged brain. Furthermore, while Asrij depletion can partially reduce the levels of pro-inflammatory cytokines, restoring them to the levels seen in young mice may depend on additional factors.

Several signaling pathways, such as NF-kB, STAT3, PI3K-Akt, NLRP3 inflammasome, and cGAS-STING, mediate neuroinflammation in the brain. Asrij promotes STAT3 and NF-κB activation in microglia to promote neuroinflammation in AD (Dongre et al., 2025). Hence, we checked the status of IL-6/STAT3 and TNF-α/NF-κB signaling pathways in the brains of aged asrij KO mice. Immunoblotting analyses showed a decline in the levels of TNF-α and phospho-NF-kB (Ser536), indicating attenuated NF-κB activation in the KO hippocampus and cortex (Figure 2C). Reduced levels of IL-6 and phospho-STAT3 (Tyr705) were also observed, suggesting decreased STAT3 activation (Figure 2D). Thus, the loss of Asrij attenuates the activation of signaling effectors known to propagate neuroinflammation in the aged mouse brain.

### Asrij depletion attenuates LPS-induced microglial activation and neuroinflammation in aged mice

**Figure 3:**
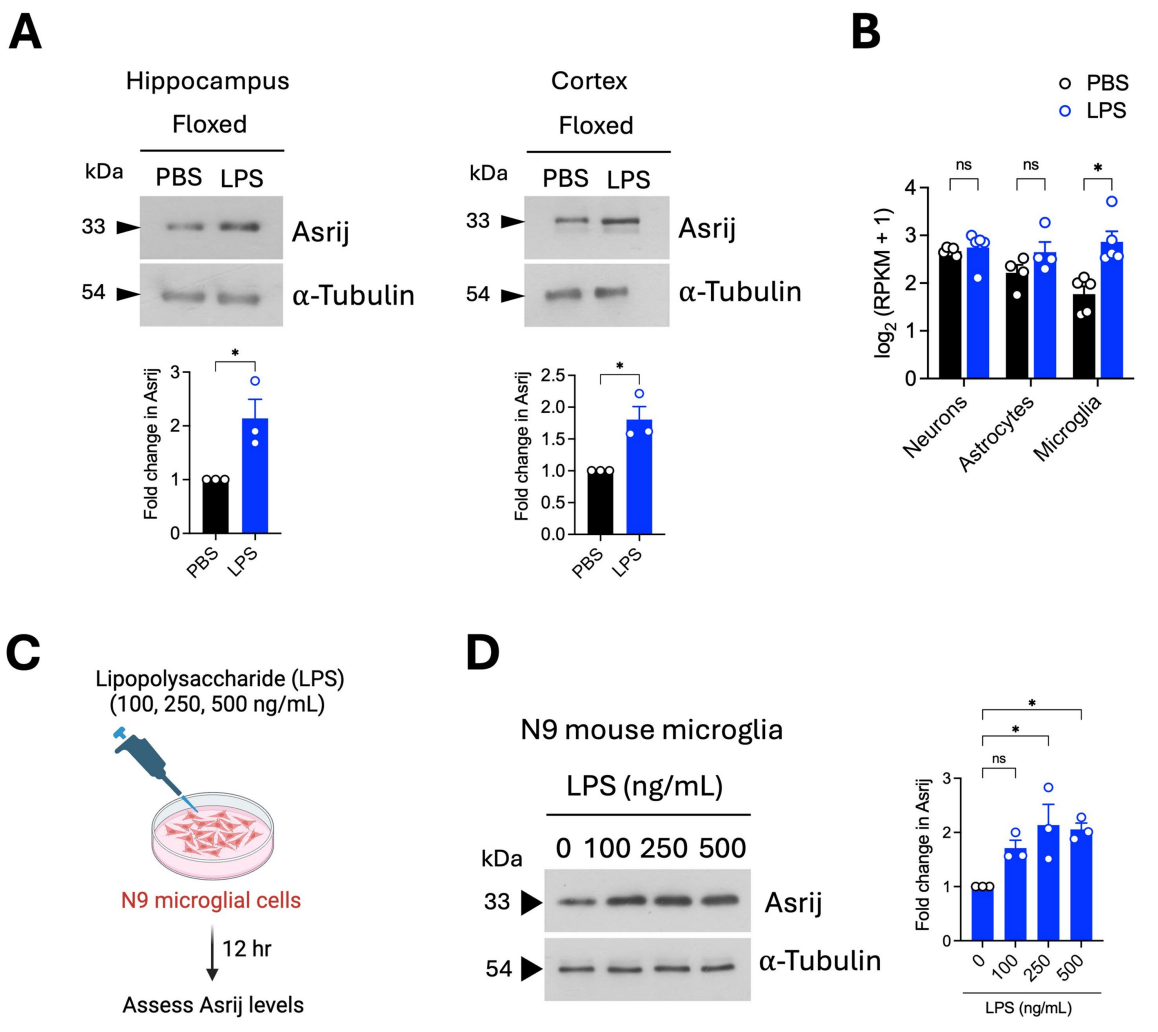
Asrij levels are increased in the mouse brain and microglia after LPS treatment. Aged (24-month-old) male mice were used in the experiments. **(A)** Immunoblot analysis of Asrij in the hippocampus and cortex of aged mice following LPS treatment (*n* = 3 mice). **(B)** Graph shows Asrij expression [log_2_ (RPKM + 1)] in neurons, astrocytes, and microglia of mice treated with PBS or LPS (*n* = 5 mice). Data are plotted from Srinivasan et al. (2016). **(C)** Schematic shows the experimental outline of the *in vitro* LPS treatment of the N9 mouse microglial cell line. **(D)** Immunoblot analysis of Asrij in N9 mouse microglial cells. α-Tubulin is the loading control. Graph shows the quantification of the fold change in Asrij protein levels normalized to the loading control (*n* = 3). Statistical significance between the experimental groups was calculated using an unpaired two-tailed Student’s t-test. Error bars denote mean ± SEM. ns—non-significant, *p < 0.05, **p < 0.01, and ***p < 0.001.

Healthy aging is characterized by increased neuroinflammatory “priming,” wherein a peripheral immune stimulus causes an exaggerated release of pro-inflammatory cytokines in the brain. This is largely ascribed to functional alterations in microglia, which are the main sources of inflammatory molecules (Norden and Godbout, 2013; Perry and Holmes, 2014). As Asrij depletion diminished the basal neuroinflammatory state, we evaluated the potential role of Asrij in controlling neuroinflammatory priming. To this end, we used an acute *in vivo* LPS injection model, which is widely used to study responses to neuroinflammation. We first evaluated the status of Asrij expression in the brain and microglia upon LPS challenge. Immunoblotting analyses showed increased Asrij protein levels in the hippocampus and cortex of LPS-treated aged *asrij* floxed mice (Figure 3A). A publicly available bulk RNA sequencing dataset showed increased Asrij transcript levels in sorted microglia upon LPS treatment (Srinivasan et al., 2016) (Figure 3B). Furthermore, increased Asrij protein levels were detected upon LPS stimulation of the N9 mouse microglial cell line (Figure 3C). This confirms that Asrij levels are elevated in the mouse brain and microglia in response to LPS.

**Figure 4:**
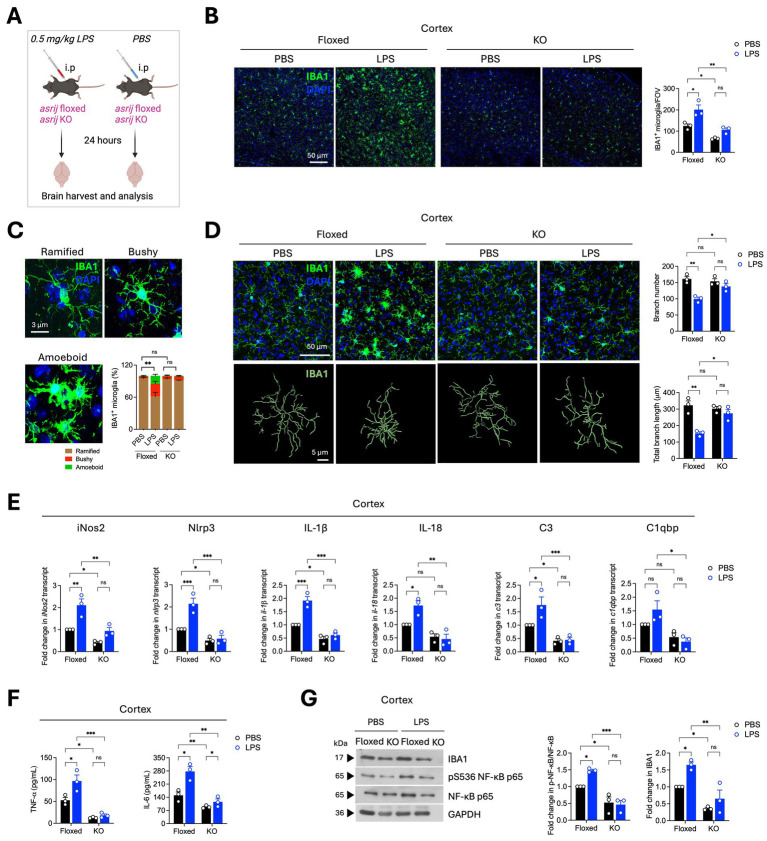
Asrij depletion abrogates LPS-induced microglial activation and neuroinflammatory responses. Aged (24-month-old) male mice were used in the experiments. **(A)** Schematic shows the experimental outline for the LPS treatment of mice. **(B)** Representative confocal images show IBA1 staining in the cortex. The graph shows the quantification of the number of IBA1^+^ microglia per field of view (FOV) of the cortex (*n* = 3 mice, 3 sections per mouse). **(C)** Representative confocal images show the typical microglial morphologies. The graph shows the quantification of the proportions of ramified, bushy, and amoeboid microglia (*n* = 3, 25 microglia per mouse) in the cortex. **(D)** Representative confocal images and Imaris-based filament rendering of IBA1 staining in the cortex. Graphs show the quantification of microglial branch number and branch length (*n* = 3, 25 microglia per mouse). **(E)** Graphs show the quantification of the fold change in normalized transcript expression in the cortex analyzed by RT-qPCR (*n* = 3 mice). GAPDH is used as a loading control. **(F)** Graphs show quantification of concentrations of TNF-α and IL-6 (pg/mL) in the cortex as determined by ELISA (*n* = 3 mice). **(G)** Immunoblots show IBA1, phospho-NF-κB (Ser536), and NF-κB levels in the cortex. GAPDH is used as a loading control. The graph shows the quantification of the fold change in normalized protein levels (*n* = 3 mice). Statistical significance between experimental groups was calculated using two-way ANOVA with Tukey’s post-hoc test. Error bars denote mean ± SEM. ns—non-significant, *p < 0.05, **p < 0.01, and ***p < 0.001.

Next, we assessed the impact of Asrij depletion on LPS-induced microglial activation and neuroinflammation (Figure 4A). In control (floxed) mice, intraperitoneal LPS administration caused an increase in microglial number, indicating microglial proliferation. In contrast, KO mice injected with LPS showed no difference in microglial numbers (Figure 4B). Microglial morphology is a standard indicator of activation state. Homeostatic/resting microglia display ramified morphology with long and thin branches. Activated microglia show a ‘bushy’ or ‘amoeboid’ morphology. LPS-treated floxed mice showed an increased proportion of bushy and amoeboid microglia. Interestingly, KO mice had a significantly reduced fraction of activated microglia (Figure 4C). Additionally, Imaris-based filament analysis showed reduced microglial branch number and length upon LPS treatment in control mice. KO microglia failed to undergo morphological transition in response to LPS (Figure 4D). Collectively, these results demonstrate that *asrij* knockout impedes LPS-induced microglial activation in aged mice.

LPS induces the activation of several inflammatory signaling cascades that lead to transcriptional upregulation of inflammatory genes (Jung et al., 2023). To probe the impact of Asrij depletion on pro-inflammatory gene expression, RT-qPCR analysis was performed. Asrij knockout significantly inhibited LPS-induced upregulation of iNos2, Nlrp3, IL-1β, IL-18, C3, and C1qbp (Figure 4E). Furthermore, ELISA revealed that KO mice failed to show an increase in the levels of TNF-*α* and IL-6 upon LPS injection, as observed in the control mice (Figure 4F). KO mice also did not exhibit increased levels of IBA1 or phosphorylated NF-κB in response to LPS, further confirming a dampened neuroinflammatory response (Figure 4G). Taken together, our results suggest that Asrij may be required for LPS-induced neuroinflammation.

## Discussion

Clinical and experimental evidence increasingly shows that neuroinflammation plays a key role in aging and age-related brain disorders. Exacerbated neuroinflammatory responses in aged individuals are primarily linked to microglial activation (Han et al., 2024; Leng and Edison, 2021). While it is well appreciated that glial “priming” is a major underlying factor for the age-associated shift in inflammatory potential of the brain, molecular regulators that govern such sensitized immune responses are not well known.

In silico analysis combined with experimental data from patients with AD and mouse models linked increased levels of Asrij/OCIAD1 to brain aging and Alzheimer’s disease. While *asrij* depletion in the AD mouse model protects from Aβ pathology by suppressing microglia-mediated neuroinflammation, the role of Asrij during normal brain aging was not explored. In this study, it is shown that normal aging is accompanied by increased microglial and astroglial activation in the hippocampus and cortex of WT mice. While *asrij* depletion did not impact glial coverage in young mice, aged *asrij* KO mice brains showed reduced numbers of microglia and astrocytes, which was accompanied by decreased neuroinflammation. This suggests that Asrij functions as a pivotal factor in maintaining the basal neuroinflammatory state in the aged brain. Accumulation of Damage-Associated Molecular Patterns (DAMPs) in the aged brain results in persistent activation of several signaling mediators, such as NF-κB, MAPK, and STAT3, among others (Davies et al., 2017; Deczkowska et al., 2017; Fonken et al., 2016). Asrij depletion resulted in reduced levels of phospho-NF-κB and phospho-STAT3, indicating that Asrij may regulate the signaling molecules that control inflammation. This likely explains the decline in the levels of inflammatory cytokines, as their production is governed by immune signaling pathways.

Chronic and low-grade neuroinflammation in aged mice increases susceptibility to infections and inflammatory stimuli. Aged rodents display exacerbated levels of inflammatory cytokines, microglial activation, and depression-like behavior upon systemic injection of LPS (Perry and Holmes, 2014). We found that *asrij* KO mice failed to display LPS-induced microglial activation and pro-inflammatory responses. This suggests that Asrij may be required to mount a neuroinflammatory response to LPS. Asrij may be involved in ‘priming,’ a microglial state of heightened sensitivity to immune stimuli, as its depletion desensitizes the aged mice to LPS challenge. Nevertheless, the molecular mechanisms by which Asrij regulates LPS-mediated neuroinflammation require further investigation. Asrij depletion leads to increased expression of genes encoding mitochondrial electron transport chain complexes and increases the mitochondrial membrane potential in AD microglia (Dongre et al., 2025). Additionally, Asrij acts as a scaffold protein to regulate several key signaling pathways related to immunity and inflammation in *Drosophila* and mice (Praveen et al., 2020). Hence, it is reasonable to speculate that similar mechanisms may operate in the context of age-related neuroinflammation.

Asrij is ubiquitously expressed in mice and humans, and thus, it is impossible to dissect the brain-specific and systemic effects of *asrij* deletion using a whole-body KO model. Previous studies have shown that *asrij* KO mice exhibit increased myeloid cells in the periphery and premature hematopoietic aging (Sinha et al., 2019, 2022). In contrast, we report reduced neuroinflammation in the aged *asrij* KO brain. Whether Asrij affects the systemic inflammatory responses warrants further investigation. Emerging evidence has shown that peripheral immune cells and blood-borne factors can affect microglia and brain inflammatory processes (Di Benedetto and Müller, 2019; Li et al., 2021; Zhang et al., 2025). As we did not perform transcardial perfusion in our experiments, it is possible that the reduced IBA1 staining observed in aged mice may be due to the reduced infiltration of peripheral myeloid cells. Thus, it is important to address whether the phenotypes we report are a direct consequence of Asrij depletion in the brain or an indirect effect of Asrij depletion in the hematopoietic system. Furthermore, the effects of reduced neuroinflammation on the cognitive behavior of *asrij* KO mice must also be assessed.

It is increasingly appreciated that microglial phenotypes and age-related neuroinflammation are sex-dependent (Delage et al., 2021; Kang et al., 2024; Lynch, 2022; Ocañas et al., 2023). Aged microglia from female mice show a stronger tendency towards a senescent and pro-inflammatory phenotype than those from male mice. Furthermore, female microglia undergo metabolic rewiring and display increased phagocytosis compared to male microglia during aging (Delage et al., 2021; Kang et al., 2024). Since our current study utilized only male mice, it is worth testing whether Asrij depletion affects glial activation and neuroinflammation differently in female mice.

Although chronic neuroinflammation during aging impairs behavior, a moderate inflammatory response is necessary to fight infections and to promote tissue repair. Whether *asrij* depletion may impair the ability to fight infections and alter susceptibility to other brain disorders merits further investigation. This will help to understand whether Asrij may be protective or detrimental during aging. Overall, our study indicates that the increased Asrij levels observed in the aged brain are vital to maintaining a basal inflammatory state and contribute to neuroinflammatory priming, which could be clinically relevant to several age-associated neurological and immune disorders.

## Data Availability

The raw data supporting the conclusions of this article will be made available by the authors, without undue reservation.
