# Supplementary material for: Asrij/OCIAD1 contributes to age-associated microglial activation and neuroinflammation in mice
Source: Front Aging Neurosci. 2025 Oct 10;17:1674136. doi: 10.3389/fnagi.2025.1674136 (PMC12549667; doi:10.3389/fnagi.2025.1674136)
Supplement: Supplementary file 1 [file Table_1.DOCX]

**Supplementary file**

**Asrij/OCIAD1 contributes to age-associated microglial activation and neuroinflammation in mice**

**Prathamesh Dongre^1^, Madhu Ramesh^2^, Thimmaiah Govindaraju^2^* and Maneesha S. Inamdar^1,3^***

^1^Molecular Biology and Genetics Unit, Jawaharlal Nehru Centre for Advanced Scientific Research (JNCASR), Bangalore 560064, Karnataka, India

^2^Bioorganic Chemistry Laboratory, New Chemistry Unit, Jawaharlal Nehru Centre for Advanced Scientific Research (JNCASR), Bangalore 560064, Karnataka, India

^3^Institute for Stem Cell Science and Regenerative Medicine (inStem), Bangalore 560065, Karnataka, India

*** Correspondence:**Thimmaiah Govindaraju ([tgraju@jncasr.ac.in](mailto:tgraju@jncasr.ac.in))

Maneesha S. Inamdar ([inamdar@jncasr.ac.in](mailto:inamdar@jncasr.ac.in); [inamdar@instem.res.in](mailto:inamdar@instem.res.in))

**Keywords: Asrij/OCIAD1, Aging, Microglia, Astrocytes, Lipopolysaccharide, Inflammatory signaling, Neuroinflammation**

**
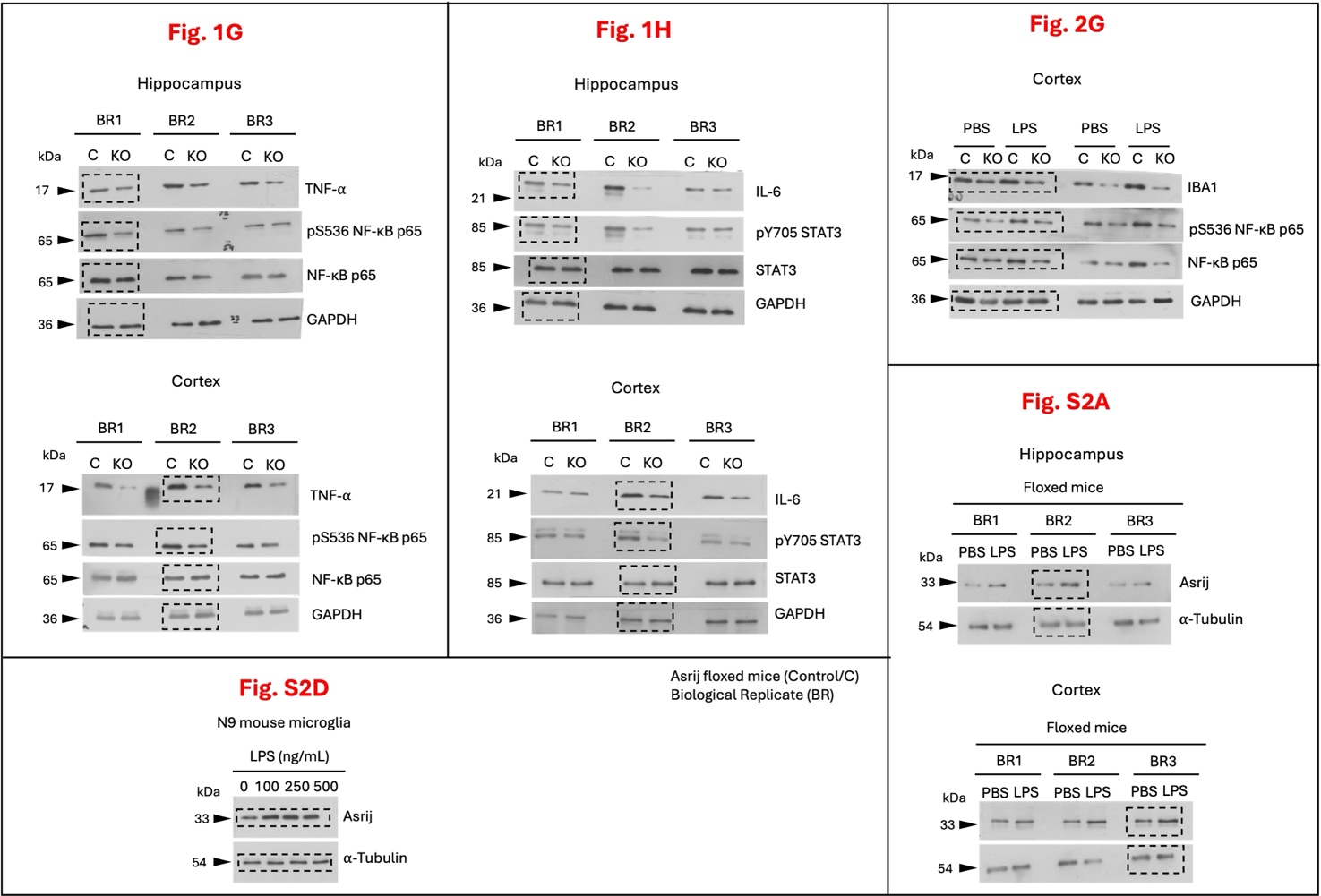
Uncropped immunoblots**
